# P-1518. In Vivo Kinetics and Transgene Expression Pattern of a Mucosal Adenovirus Vaccine Vector

**DOI:** 10.1093/ofid/ofaf695.1702

**Published:** 2026-01-11

**Authors:** Alexander C Vostal, Dawid Maciorowski, Ceanna Cooney, Peyton M Roeder, Apisit Patamawenu, Rachel Roenicke, Anna Hostal, Freya van’t Veer, Mitra Harrison, Breanna Kim, Guangping Liu, Kening Wang, Mark Connors, Jeffrey I Cohen

**Affiliations:** Laboratory of Infectious Diseases, National Institute of Allergy and Infectious Diseases, National Institutes of Health, Bethesda, Maryland; Laboratory of Infectious Diseases, National Institute of Allergy and Infectious Diseases, National Institutes of Health, Bethesda, Maryland; HIV-Specific Immunity Section of the Laboratory of Immunoregulation, National Institute of Allergy and Infectious Diseases, National Institutes of Health, Bethesda, Maryland; HIV-Specific Immunity Section of the Laboratory of Immunoregulation, National Institute of Allergy and Infectious Diseases, National Institutes of Health, Bethesda, Maryland; HIV-Specific Immunity Section of the Laboratory of Immunoregulation, National Institute of Allergy and Infectious Diseases, National Institutes of Health, Bethesda, Maryland; HIV-Specific Immunity Section of the Laboratory of Immunoregulation, National Institute of Allergy and Infectious Diseases, National Institutes of Health, Bethesda, Maryland; Laboratory of Infectious Diseases, National Institute of Allergy and Infectious Diseases, National Institutes of Health, Bethesda, Maryland; HIV-Specific Immunity Section of the Laboratory of Immunoregulation, National Institute of Allergy and Infectious Diseases, National Institutes of Health, Bethesda, Maryland; HIV-Specific Immunity Section of the Laboratory of Immunoregulation, National Institute of Allergy and Infectious Diseases, National Institutes of Health, Bethesda, Maryland; HIV-Specific Immunity Section of the Laboratory of Immunoregulation, National Institute of Allergy and Infectious Diseases, National Institutes of Health, Bethesda, Maryland; Laboratory of Infectious Diseases, National Institute of Allergy and Infectious Diseases, National Institutes of Health, Bethesda, Maryland; Laboratory of Infectious Diseases, National Institute of Allergy and Infectious Diseases, National Institutes of Health, Bethesda, Maryland; HIV-Specific Immunity Section of the Laboratory of Immunoregulation, National Institute of Allergy and Infectious Diseases, National Institutes of Health, Bethesda, Maryland; Laboratory of Infectious Diseases, National Institute of Allergy and Infectious Diseases, National Institutes of Health, Bethesda, Maryland

## Abstract

**Background:**

Many viral pathogens are transmitted by mucocutaneous infection, but only a small portion of licensed vaccines are administered via the mucosa. A recombinant adenovirus serotype 4 (Ad4) vector expressing influenza H5 haemagglutinin has demonstrated the ability to safety induce long lasting mucosal immune responses. To better understand the pattern of transgene expression and kinetics of the Ad4 vector, we designed a recombinant Ad4 vector that expresses Luciferase (Ad4-Luc). An in vivo imaging system (IVIS) was used to track Luc expression after different routes of administration in a murine model.

**Methods:**

Luc was cloned into the E3 region of Ad4 and the virus was propagated in A549 cells. Female BALB/c mice were inoculated with Ad4-Luc intramuscularly (IM), intranasally (IN), intravaginally (Ivag), or via gastric gavage. Luciferase expression after Ad4-Luc inoculation was quantitated by radiance via IVIS after injection of luciferin in live mice and by incubating tissues with luciferin at necropsy. Virus replication and shedding of Ad4 DNA were compared in mice that received cidofovir or no antiviral.

**Results:**

Local Luc expression was observed after IM, IN or Ivag Ad4-Luc inoculation of mice. IN inoculation produced the highest level of Luc expression with radiance in the snout detected from 7 hours to 13 days after inoculation, peaking on day 4, and was less intense with subsequent inoculations. Luc expression after IN inoculation was observed ex vivo in the lungs, kidney and rectum. Cidofovir decreased the level of nasal shedding of viral DNA, but not Luc expression.

**Conclusion:**

Intranasal inoculation of mice with Ad4-Luc resulted the highest levels of local transgene expression that lasted for nearly 2 weeks. The recombinant Ad4 vector should be considered as a candidate for future mucosal vaccination strategies.

**Disclosures:**

All Authors: No reported disclosures

